# Prosocial lie-telling in preschoolers: The impacts of ethnic background, parental factors, and perceived consequence for the partner

**DOI:** 10.3389/fpsyg.2023.1128685

**Published:** 2023-03-30

**Authors:** Roksana Dobrin-De Grace, Lili Ma

**Affiliations:** Department of Psychology, Toronto Metropolitan University, Toronto, ON, Canada

**Keywords:** prosocial lie-telling, ethnic background, collectivist orientation, parenting style, positive face

## Abstract

This study explored prosocial lie-telling behavior in 4- to 5-year-old children from two ethnic groups: European Canadian (*n* = 49; excluding Eastern European Canadian) and Chinese Canadian (*n* = 45). Children completed an online experiment involving two real-life politeness situations. In the first situation, children were asked whether they thought someone with a red mark on their face looked okay for a photo or a Zoom party (Reverse Rouge Task). In the second situation, upon hearing the researcher’s misconception about two pieces of artwork, children were asked whether they agreed with the researcher (Art Rating Task). Parents completed questionnaires that measured their levels of collectivist orientation and parenting styles. Contrary to our hypotheses, the likelihood of children telling a prosocial lie did not vary as a function of their ethnic group or the presence of a perceived consequence for the partner, nor was it predicated by parental collectivist orientation. Interestingly, prosocial liars were more likely to have authoritative parents, whereas blunt-truth tellers were more likely to have permissive parents. These findings have important implications for the ways in which certain parenting styles influence the socialization of positive politeness in children. In addition, the similar rates of prosocial lying across the two ethnic groups suggest that children who are born and raised in Canada may be much more alike than different in their prosocial lie-telling behavior, despite coming from different ethnic backgrounds.

## 1. Introduction

Prosocial lying, or telling lies to benefit others, is a ubiquitous social behavior and emerges early in development (e.g., Talwar et al., 2002; Warneken and Orlins, 2015). People may lie to others for various prosocial purposes, ranging from being polite, protecting someone’s feelings, avoiding interpersonal conflicts, to increasing group cohesion (e.g., Bryant, 2008; Levine and Lupoli, 2022). When viewed through the lens of Brown and Levinson’s politeness theory, prosocial lying is a positive politeness strategy that seeks to avoid offending the receiver’s positive face or their desire for their self-image to be appreciated and accepted by others (Brown et al., 1987).

Children are able to tell prosocial lies from early on. For example, children as young as 3 would tell an adult that they looked good for a picture, despite having a red mark on their face (Talwar and Lee, 2002). Upon receiving a disappointing gift, 3-year-olds would lie to make the gift giver believe that the gift was desirable (Talwar et al., 2007). As children get older, the frequency of their prosocial lying increases (e.g., Broomfield et al., 2002; Warneken and Orlins, 2015), and they become adept at embellishing their prosocial lies with other prosocial behaviors to strengthen their credibility, such as excessive smiling or explaining why an undesirable gift was in fact desirable (Talwar et al., 2007).

Previous studies have shown some cultural and ethnic group differences in children’s moral evaluations of prosocial lies (e.g., Lee et al., 1997; Fu et al., 2010; Cameron et al., 2012; Chiu Loke et al., 2014). For instance, Chiu Loke et al. (2014) found that 7- to 11-year-old American children considered it more appropriate to lie to a teacher about a friend’s minor transgression than Japanese peers did. In another study, Cameron et al. (2012) found that at age 11, Chinese children had the most favorable views of modest lies, followed by Chinese Canadian children, with European Canadian children holding the least favorable views of modest lies. These findings may be explained by different emphasizes that collectivist versus individualist cultures place on socialization goals with reference to social responsibility, compliance to authority figures, and respecting modesty (Fu et al., 2010; Cameron et al., 2012; Chiu Loke et al., 2014).

There is also evidence for the influence of parenting on children’s prosocial lie-telling. Popliger et al. (2011) found a positive association between authoritative parenting style and 4- to 12-year-olds’ tendency to tell a prosocial lie when asked how they liked a disappointing gift. In particular, the parents of children who told a prosocial lie were more authoritative in their parenting styles than the parents of children who told the truth. No association was found between children’s prosocial lying and authoritarian or permissive parenting styles. In politeness situations such as receiving a disappointing gift, telling prosocial lies to be polite or to avoid hurting the partner’s feelings is a social convention in most societies. It is an important social skill that authoritative parents may value to a greater extent in the socialization of their children than parents with other parenting styles (Popliger et al., 2011).

Taken together, the studies described above suggest that children’s prosocial lie-telling behavior is subject to influence from their cultural or ethnic backgrounds and parenting styles. A few questions remain open and warrant further examination. First, previous work on cultural or ethnic group differences in prosocial lying has focused primarily on children’s moral evaluations of a protagonist’s behaviors in hypothetical scenarios. It has yet to be examined whether there are cultural or ethnic group differences in children’s *actual prosocial lying* in real-life situations, especially given that an individual’s moral judgment of others may not always align with their own moral behavior (e.g., Saltzstein, 1994). Second, the observed cultural or ethnic differences were often interpreted as expressions of different socialization goals endorsed by collectivist versus individualist cultures. Yet, collectivist versus individualist orientations in parents from different cultural or ethnic backgrounds have not been directly measured to support this interpretation. Third, existing research on the relation between children’s actual prosocial lying and parenting styles is limited to one politeness situation, namely receiving a disappointing gift. It has yet to be examined whether parenting styles play a role in children’s prosocial lying in other politeness situations.

The present study aims to extend previous research by addressing these gaps. In particular, we explored children’s prosocial lying in real-life situations in relation to their ethnic background, parental collectivistic orientation, and parenting style. In addition to these social variables, we also examined the effect of a perceived consequence for the partner. It has been found that children consider it more appropriate to tell the truth rather than a prosocial lie when the truth could be helpful to the partner (Heyman et al., 2020) or could spare the partner from a serious consequence (e.g., Ma et al., 2011; Lau et al., 2013; Fu et al., 2016). As such, the presence of a perceived consequence for the partner may motivate children to favor truth-telling over prosocial lying.

Canadian preschoolers from two ethnic groups—European Canadian (excluding Eastern European Canadian) and Chinese Canadian—were tested in this study. Their prosocial lie-telling behavior was assessed online in two real-life politeness situations where they had to be sensitive to an adult’s positive face. In a Reverse Rouge Task, children were asked whether they thought an adult with a red mark on their chin looked okay for a photo (“no consequence” condition) or for a big Zoom party with friends and family (“consequence” condition). In an Art Rating Task, upon hearing an adult’s misconception about two pieces of artwork (“no consequence” condition) or about submitting her artwork to an art contest (“consequence” condition), children were asked whether they shared the adult’s opinion. For both politeness situations, based on previous findings described above, we hypothesized that:

*H1*: Chinese Canadian children would be more likely than European Canadian children to tell a prosocial lie.

*H2*: A higher parental score in collectivist orientation would be associated with a greater likelihood of children telling a prosocial lie.

*H3*: A higher score in authoritative parenting style would be associated with a greater likelihood of children telling a prosocial lie.

*H4*: Children would be less likely to tell a prosocial lie when there is a potential consequence for the partner than when there is no such consequence.

## 2. Materials and methods

### 2.1. Participants

Participants were 94 Canadian children aged 4–5: 49 European Canadian (*M* = 4 years 10 months; 27 girls), and 45 Chinese Canadian (*M* = 4 years 11 months; 29 girls). We conducted an *a priori* power analysis using G*Power (Faul et al., 2009) based on the recommendations by Hsieh et al. (1998) and Chen et al. (2010). The results suggested that 92 participants would be needed for 80% power with an odds ratio of 2.5 (small to medium effect size). The European Canadian children came from households where English is the primary spoken language and the parents self-identified as White but not Eastern European. The Chinese Canadian children came from households where Mandarin or Cantonese is the primary spoken language and the parents self-identified as Chinese. Children were recruited from two metropolitan areas in Canada (*n* = 88 and 6, respectively). See Supplementary Table S1 for participant demographics. This study received ethics approval from the Toronto Metropolitan University.

### 2.2. Stimuli and parental measures

Each child completed two experimental tasks online. The parent completed three questionnaires *via* Qualtrics: a demographics questionnaire, the Individualism and Collectivism Scale (INDCOL; Singelis et al., 1995; Cronbach’s alpha = 0.73 to 0.82; Cozma, 2011), and the Parenting Styles and Dimensions Questionnaire (PSDQ; Baumrind, 1971; Cronbach’s alpha = 0.73 to 0.82, Robinson et al., 1995).

#### 2.2.1. Reserve Rouge Task

The first task was modeled after Talwar and Lee (2002). In this task, a female researcher had a red lipstick smear on her chin and asked children if she looked okay for a photo or a Zoom party. The experimental session began with the researcher introducing herself. Then she put on a one-minute educational video about kittens for the child to watch and turned off her camera. While off camera, she applied a lipstick smear on her chin. After the video was complete, she returned on camera and asked the child if they enjoyed watching the video. The next step differed across conditions. In the “no consequence” condition, the researcher simply asked the child if she looked good for a photo. In the “consequence” condition, the researcher first told the child that she was going to a big Zoom party with her friends and family right after their video call. She then asked the child if she looked okay to attend the party. In both conditions, a prosocial lie was noted if the child responded verbally or nonverbally that the researcher looked okay for a photo or for the party.

#### 2.2.2. Art Rating Task

The second task was modeled after Warneken and Orlins (2015). In this task, the researcher showed the child three pairs of artwork: one pair of photographs for the baseline trial and two pairs of drawings for the two test trials (see Supplementary Figure S1). Within each pair, one piece (A) was better done than the other (B). The baseline trial was conducted first, followed by two test trials. The beginning of each trial differed across conditions: In the “no consequence” condition, the researcher told the child that she would like their opinion on some of her artwork, whereas in the “consequence” condition, the researcher told the child that she would like their opinion on some of her artwork that she was preparing for an art contest. Afterwards, the researcher told the child that she thought A was better than B (baseline trial) or B was better than A (test trials) and asked the child if they agreed. A prosocial lie was noted if the child agreed with the researcher verbally or nonverbally.

The three pairs of artwork were selected from a larger sample based on feedback from 15 adults. To avoid making children suspect that the intent was to deceive them, the artwork chosen as the final stimuli could not be too perceptibly different in quality. That is, the “worse” artwork had to have some merit to them, otherwise the politeness scenario could have come across to children as a joke. An additional 16 children aged 4–5 completed a manipulation check for the drawings used for the test trials, where they were asked which drawing within each pair they thought was the better one. For both pairs of drawings, 88% of the children (14/16) judged A (i.e., the one that was better done) to be better than B.

#### 2.2.3. Parental measures

The INDCOL measures if the test-taker is more individualistically or collectivistically oriented. It provides four scores: Horizontal Individualism (HI), Vertical Individualism (VI), Horizontal Collectivism (HC), and Vertical Collectivism (VC). The vertical dimension refers to an acceptance with hierarchy in society, whereas the horizontal dimension refers to an other-regarding orientation or an egalitarian outlook (Cozma, 2011). The PSDQ measures parenting styles and attitudes regarding parenting. It provides three scores: (1) authoritative parenting style, exhibiting high warmth and high demandingness, (2) authoritarian parenting style, exhibiting low warmth and high demandingness, and (3) permissive parenting style, exhibiting low warmth and low demandingness (Robinson et al., 1995).

### 2.3. Procedure

Prior to the experiment, parents completed the questionnaires *via* Qualtrics. On the day of the experiment, the child participated online *via* Zoom or Google Meet. Each child was randomly assigned into one of the two conditions. The video call began with a warm-up phase with two females—the researcher who administered the experimental tasks and a research assistant who was off camera during the experiment. Child assent was obtained before the procedure began. With parental consent and child assent, the session was video-recorded. The order of the two tasks was counterbalanced. Between the tasks, the child watched a one-minute educational video. At the end of the experiment, the researcher debriefed the child and thanked them for their help. Each child received an electronic certificate as an appreciation and a $5 e-gift card.

## 3. Results

Preliminary analyses showed no gender differences in children’s prosocial lying or parental measures, so gender was not included in the main analyses reported below. The alpha level used to determine significance is *p* < 0.05 (two-tailed). For each experimental task, children’s prosocial lie-telling behavior was first compared to chance responding and then analyzed using a logistic regression with ethnic background, condition, and the parental measures as predictors. The raw data set is included as a Supplementary file.

### 3.1. Parental measures

The parents of the Chinese Canadian children scored significantly higher in their authoritarian parenting style and vertical individualist orientation than the parents of the European Canadian children, *t*(92) = 3.67, *p* < 0.001, Cohen’s *d* = 0.47, and *t*(91) = 4.34, *p* < 0.001, Cohen’s *d* = 1.19, respectively (independent-samples *t*-tests). The two groups were comparable on the other parental measures (see Table 1).

**Table 1:** Mean scores on the PSDQ and INDCOL by ethnic group.

Group	*N*	PSDQ			INDCOL			
		Authoritativeness	Authoritarianism	Permissiveness	Vertical individualism	Horizontal individualism	Vertical collectivism	Horizontal collectivism
European Canadian	49	4.32 (0.35)	1.65 (0.32)	2.31 (0.49)	4.03 (1.16)	6.28 (1.32)	6.40 (1.14)	7.42 (0.93)
Chinese Canadian	44	4.33 (0.37)	2.00 (0.60)^***^	2.51 (0.68)	5.10 (1.21)^***^	5.98 (1.11)	6.59 (1.36)	7.52 (0.84)

^***^p < 0.001 in comparison to the European Canadian group. SDs in parentheses. PSDQ scores are out of 5. INDCOL scores are out of 9. In this table and Table 2, data from one participant were included for Parental Authoritativeness and Authoritarianism only due to missing responses to the other parental measures.

### 3.2. Reverse Rouge Task

**Figure 1:**
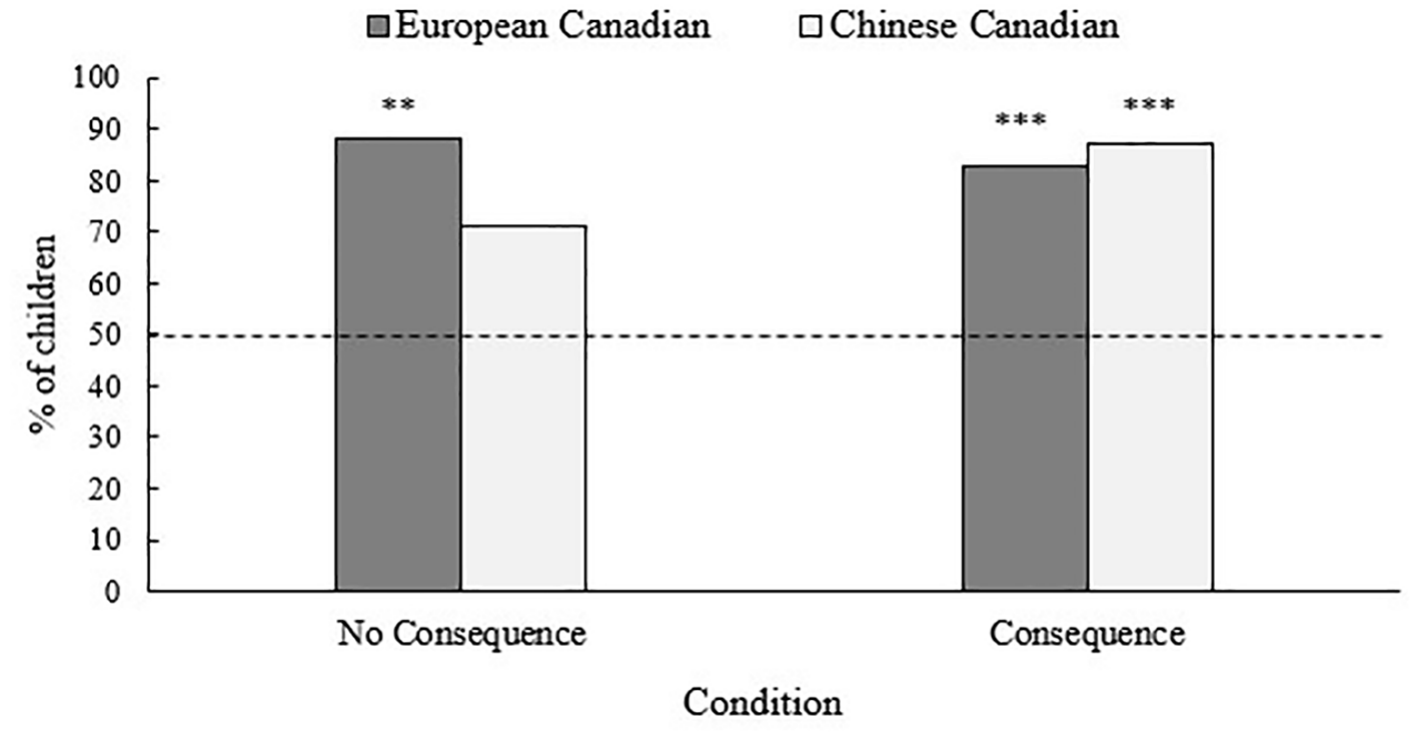
Percentage of children telling prosocial lies (by condition) in the Reverse Rouge Task. ***p < 0.001, **p < 0.01 as compared to chance expectation (50%; dashed line).

Overall, 84% of the children lied in the Reverse Rouge Task, by telling the researcher that she looked good for a photo or for a Zoom party. One-sample binomial tests indicated that the percentage of the European Canadian children who lied was significantly greater than would be expected by chance (50%), 88%, *p* < 0.001 (“no consequence” condition), and 83%, *p* = 0.002 (“consequence” condition), respectively. The percentage of the Chinese Canadian children who lied significantly exceeded chance expectation in the “consequence” condition (87%, *p* < 0.001) but not in the “no consequence” condition (71%, *p* = 0.078). See Figure 1.

A binary logistic regression showed that authoritative parenting style (*B* = 2.13, Wald = 4.17, *p* = 0.041) and permissive parenting style (*B* = −1.43.13, Wald = 4.14, *p* = 0.042) were significant predictors of children’s prosocial lying: Children who told a prosocial lie, relative to children who told the blunt truth, had parents with higher scores in authoritative parenting style and lower scores in permissive parenting style. The effects of ethnic background, condition, and the other parental variables were not significant (see Table 2).

**Table 2:** Children’s prosocial lying in relation to all variables of interest: Logistic regression results by task.

Predictor	*B/*Estimate	S.E.	Wald	*df*	Sig.	Exp(*B*)	95% CI for Exp(B) [LL, UL]
**Reverse Rouge Task (N = 92)**							
Ethnic group	−1.101	0.960	1.315	1	0.251	0.332	[0.05, 2.18]
Condition	−0.512	0.912	0.315	1	0.575	0.599	[0.10, 3.58]
Ethnic group × condition	1.615	1.314	1.511	1	0.219	5.027	[0.38, 65.98]
Authoritativeness	2.130	1.043	4.171	1	0.041*	8.414	[1.09, 64.96]
Authoritarianism	0.109	0.812	0.018	1	0.893	1.116	[0.23, 5.48]
Permissiveness	−1.431	0.703	4.144	1	0.042*	0.239	[0.06, 0.95]
Horizontal individualism	0.273	0.284	0.919	1	0.338	1.313	[0.75, 2.29]
Vertical individualism	0.548	0.338	2.638	1	0.104	1.730	[0.89, 3.35]
Horizontal collectivism	−0.711	0.483	2.164	1	0.141	0.491	[0.19, 1.27]
Vertical collectivism	−0.040	0.302	0.017	1	0.895	0.961	[0.53, 1.74]
(Constant)	−1.993	6.350	0.099	1	0.754	0.136	
							*pseudo R^2^* = 0.265
**Art Rating Task (N = 93)**							
Zero prosocial lies	3.377	4.168	0.657	1	0.418		[−4.79, 11.55]
One prosocial lie	4.504	4.181	1.161	1	0.281		[−3.69, 12.70]
Authoritativeness	1.353	0.662	4.177	1	0.041*		[0.06, 2.65]
Authoritarianism	−0.672	0.540	1.546	1	0.214		[−1.73, 0.39]
Permissiveness	0.081	0.413	0.038	1	0.845		[0.73, 0.89]
Horizontal individualism	0.098	0.171	0.327	1	0.568		[−0.24, 0.43]
Vertical individualism	0.312	0.196	2.533	1	0.112		[−0.07, 0.70]
Horizontal collectivism	−0.357	0.259	1.901	1	0.168		[−0.86, 0.15]
Vertical collectivism	−0.026	0.174	0.023	1	0.880		[−0.37, 0.31]
Ethnic group	0.650	0.483	1.812	1	0.178		[−0.30, 1.60]
Condition	−0.115	0.417	0.076	1	0.782		[−0.93, 0.70]
							*pseudo R^2^* = 0.164

A binary logistic regression was used for the Reverse Rouge Task. An ordinal regression was used for the Art Rating Task. B represents unstandardized regression weights. S.E. represents standard error. LL and UL indicate the lower and upper limits of a confidence interval, respectively. For the Art Rating Task, the reference category is “two prosocial lies.” *p < 0.05.

### 3.3. Art Rating Task

**Figure 2:**
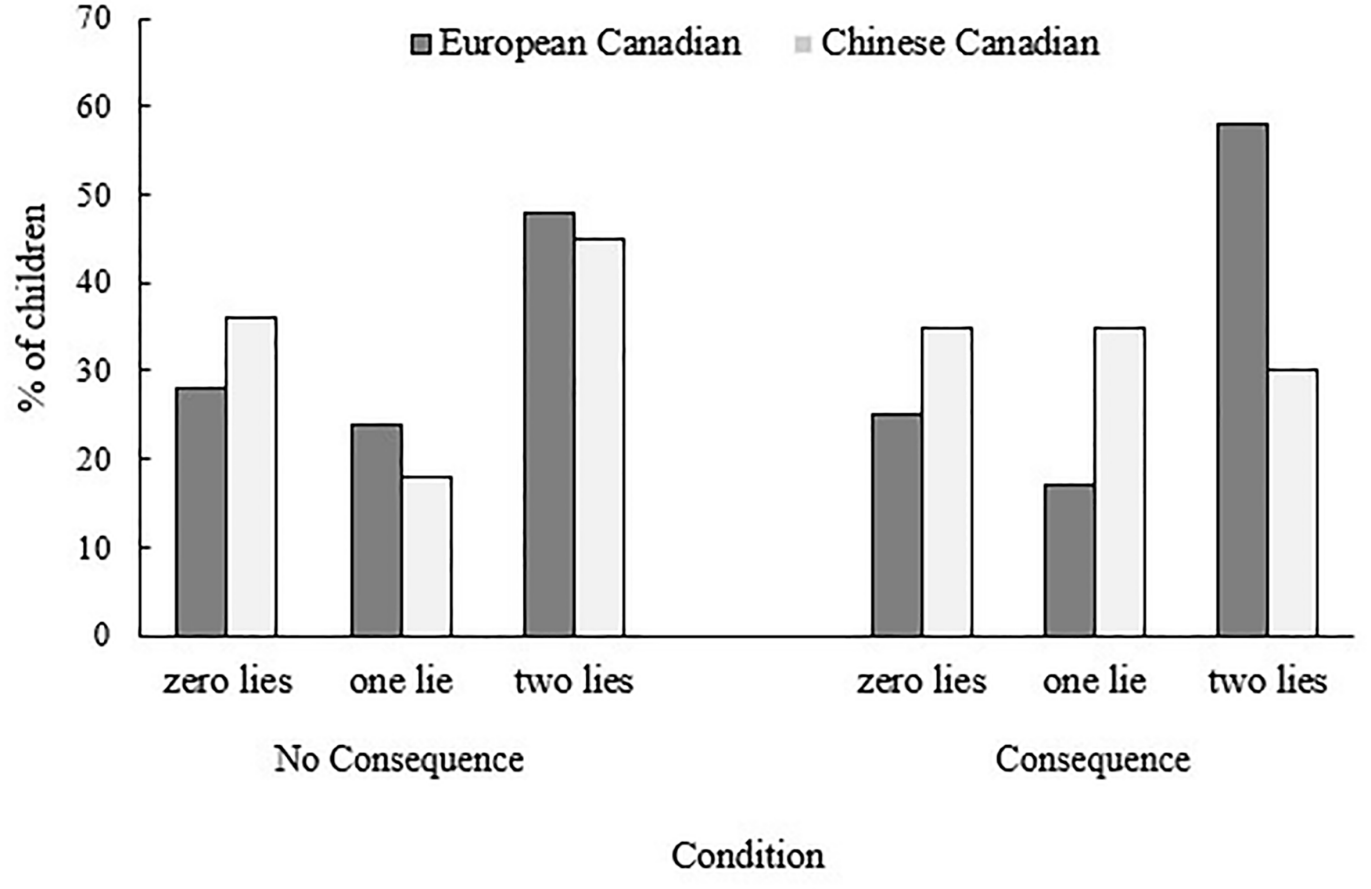
Percentage of children telling zero lies, one lie, or two lies in the Art Rating Task (by condition).

Overall, children told a prosocial lie 57.4% of the time in the Art Rating Task, by agreeing with the researcher’s misconception. Figure 2 shows the percentage of children telling 0, 1, or 2 lies across the two test trials, by condition. The number of lies was ordinal and not normally distributed (*p* < 0.001, one-sample Kolmogorov–Smirnov test). Therefore, we conducted one-sample Wilcoxon signed-rank tests to compare the median of the number of lies told by children to a hypothesized median of 1 (i.e., chance expectation). No significant differences were found across ethnic groups or conditions.

An ordinal logistic regression indicated that authoritative parenting style was a significant predictor of children’s prosocial lying, *B* = 1.35, Wald = 4.18, *p* = 0.041. The effects of ethnic background, condition, and the other parental variables were not significant (see Table 2).

### 3.4. Comparing and combining data across tasks

A cross-task comparison revealed that children who lied in the Reverse Rouge Task, relative to children who told the truth, told more prosocial lies in the Art Rating Task, *t*(91) = 2.13, *p* = 0.036, Cohen’s *d* = 0.85.

For exploratory purposes, we combined the data across tasks and examined children’s prosocial lying as a repeated measure. For the Art Rating Task, the number of prosocial lies told by children was dichotomized into 0 (zero lies) and 1 (1–2 lies). With children’s performance across tasks as a repeated measure (binomial distribution), we conducted a Generalized Linear Model using Generalized Estimating Equations (GEE). The results were consistent with those of the logistic regressions: authoritative parenting style was a significant predictor of children’s prosocial lying, *B* = 1.74, Wald = 10.05, *p* = 0.002. No other significant effect was found (see Supplementary Table S2).

## 4. Discussion

The present study examined 4- to 5-year-old children’s prosocial lying behavior in relation to their ethnic background, parental collectivist orientation, parenting style, and perceived consequences for the partner. In a video call, when asked whether an adult with a noticeable lipstick smear on her chin looked good for a photo or for a big Zoom party with family and friends, 84% of the children told a prosocial lie by saying that the adult looked good, comparable to the finding (89%) in Talwar and Lee (2002). When asked about their opinion on two pieces of artwork, children told a prosocial lie by agreeing with the adult’s misconception about 57.4% of the time.

Contrary to our first hypothesis (H_1_), in both politeness situations, the rate of children’s prosocial lying did not differ significantly across ethnic groups. This lack of group difference in a Canadian context might have something to do with the homogeneity of our sample. Although the parents of the Chinese Canadian children scored higher in their authoritarian parenting style and vertical individualist orientation than the parents of the European Canadian children, these two groups were comparable on measures of collectivist orientation and authoritative parenting style, two variables that might be particularly relevant to cultural or ethnic differences in the socialization of politeness. Therefore, there might not have been marked differences in parental socialization across the two ethnic groups, resulting in comparable rates of prosocial lying in European Canadian and Chinese Canadian children.

The present data also did not support our second hypothesis that parental collectivist orientation would be positively associated with children’s prosocial lying (H_2_). This finding appears inconsistent with previous work that has found a positive correlation between Chinese children’s favorable attitudes toward telling modest lies and their parents’ collectivist orientation (e.g., Fu et al., 2010). One possible explanation for this discrepancy is that parental collectivist orientation may not impact different types of prosocial lying in equal measure (e.g., white lies in our study versus modest lies in previous work). We also speculate that this discrepancy may arise, at least in part, out of the divergence between moral judgment and behavior. Moral judgment is often made from the perspective of an observer after a behavior has already occurred, whereas moral behavior occurs before the individual makes a decision on how to act in a moral situation from the perspective of the self. Thus, self-interest is more likely to be at risk in moral behavior than in judgment (Saltzstein, 1994). Because of this judgment-behavior divergence, it is possible that parental collectivist orientation may influence children’s moral judgment of prosocial lying, but may not be a strong enough influence on children’s *actual* prosocial lying. This explanation is tentative and requires further examination.

Our third hypothesis (H_3_) was supported, in that parental authoritativeness was positively associated with the likelihood of children’s prosocial lying. Since authoritative parents value socio-emotional development highly as a parenting goal (Pearson and Rao, 2003), it follows that children of authoritative parents may be more emotionally in tune with the consequences of their behaviors: In the Reverse Rouge Task, they might have felt uncomfortable putting the adult in a situation that would make her feel embarrassed or lose face; in the Art Rating Task, they might have acted in a more agreeable manner in order to avoid conflict or disappointing the adult. Supporting this explanation, previous work demonstrates that authoritative parents valued the development of effective social skills and engaged in practices that fostered their children’s emotional competence in a social context (e.g., Chan et al., 2009). Relatedly, Mojdehi et al. (2020) reported that greater maternal use of induction—a practice commonly adopted by authoritative parents—was associated with more positive evaluations of politeness lies in Persian and Canadian children.

A novel finding emerged in our study: in the Reverse Rouge Task, children who told a prosocial lie, relative to children who told the blunt truth, had parents with lower scores in permissive parenting style. That is, children with more permissive parents were more likely to tell the researcher that she did not look okay for the photo or for the Zoom party. Although conjectural, it is possible that more permissive parents allow certain behaviors from their children more readily, such as voicing opinions more frankly toward others. This interpretation remains tentative and requires further examination.

Finally, there was no support for our fourth hypothesis of a condition effect (H_4_). In both tasks, the rate of children’s prosocial lying was comparable across conditions. That is, the possibility of a social consequence for the researcher did not lead to more truth-telling in children, which is inconsistent with previous findings that children consider it more appropriate to tell the truth rather than a prosocial lie when the truth could spare the partner from a serious consequence (e.g., Ma et al., 2011; Fu et al., 2016). As discussed earlier, this discrepancy may have something to do with the divergence between moral judgment and behavior. Previous work primarily examined moral judgments, where children were asked for their opinion on a character’s behavior in hypothetical scenarios. In the present study, children were put in real-life politeness situations where they had to interact with an adult, which might have increased the social pressure on children and made them hesitant to tell the truth, even when there was a potential social consequence for the partner.

Overall, the present research provides evidence that an authoritative parenting style is positively associated with children’s prosocial lie-telling behavior. Contrary to our hypotheses, there is no evidence for the influence of ethnic background, parental collectivist orientation, and perceived consequence for the partner on Canadian preschoolers’ prosocial lying across two different politeness situations. It is important to note that, while we recruited children from households where both parents were of the same ethnicity, a Canadian upbringing might “override” other cultural influences and has a strong effect on children’s socialization. It is also possible that, over time, globalization has led to more similarities than differences across cultures and ethnic groups. To address this limitation, future work can compare children in China and other East Asian countries with Canadian children from different ethnic groups, which will provide a more nuanced understanding of how cultural and/or ethnic group backgrounds might impact children’s prosocial lying.

Future work can also examine possible group differences in children’s prosocial lying from the framework of cultural tightness–looseness (Gelfand et al., 2006). Tight cultures have stronger social norms and lower tolerance of socially deviant behaviors than loose cultures (Gelfand et al., 2011). It has been found that individuals in tight cultures, relative to individuals in loose cultures, are higher on compliance behavior or deference to authority and less likely to behave in accordance with their personal values (Elster and Gelfand, 2021). Based on these findings, it is possible that children in tight cultures would be more likely to tell prosocial lies to an authority figure than children in loose cultures, especially in situations where truth-telling might threaten the authority figure’s positive face.

In the present study, prosocial lying was viewed as more favorable than truth telling in politeness situations. However, in light of previous findings on the effects of stochastic versus deterministic feedback on performance (e.g., Hentschel et al., 2022), prosocial lying involves providing false feedback that may not always be in the partner’s best interest, whereas truth telling involves providing accurate feedback that can be more valuable. In our study, the children who told the truth might have done so with the intention of providing honest feedback to spare the partner from a potentially negative consequence. Future work can address this possibility by probing into the intentions underlying children’s prosocial lying versus truth telling in politeness situations.

In summary, the present data shed light on the associations between different parenting styles and children’s prosocial lying. We found that prosocial liars were more likely to have authoritative parents and that blunt-truth tellers were more likely to have permissive parents. These findings have important implications for the ways in which certain parenting styles influence the socialization of positive politeness in children. In addition, the similar rates of prosocial lying across the two ethnic groups suggest that children who are born and raised in Canada may be much more alike than different in their prosocial lie-telling behavior, despite coming from different ethnic backgrounds.

## Data availability statement

The original contributions presented in the study are included in the article/Supplementary material, further inquiries can be directed to the corresponding authors.

## Ethics statement

The studies involving human participants were reviewed and approved by the Toronto Metropolitan University Research Ethics Board. Written informed consent to participate in this study was provided by the participants’ legal guardian/next of kin.

## Author contributions

RD-DG and LM designed the study, analyzed the data, and wrote the manuscript. RD-DG performed the study. All authors contributed to the article and approved the submitted version.

## Funding

This research was funded by a Jackman Foundation Psychology Research Excellence Award from Toronto Metropolitan University. The preparation of this article was supported with a grant provided by the Office of the Dean of Arts, Toronto Metropolitan University.

## Conflict of interest

The authors declare that the research was conducted in the absence of any commercial or financial relationships that could be construed as a potential conflict of interest.

## Publisher’s note

All claims expressed in this article are solely those of the authors and do not necessarily represent those of their affiliated organizations, or those of the publisher, the editors and the reviewers. Any product that may be evaluated in this article, or claim that may be made by its manufacturer, is not guaranteed or endorsed by the publisher.

## References

[ref1] Baumrind D. (1971). Current patterns of parental authority. Dev. Psychol. Monogr. 4, 1–103. doi: 10.1037/h0030372

[ref2] Broomfield K. A. Robinson E. J. Robinson W. P. (2002). Children's understanding about white lies. Br. J. Dev. Psychol. 20, 47–65. doi: 10.1348/026151002166316

[ref3] Brown P. Levinson S. C. Levinson S. C. (1987). Politeness: Some universals in language usage (*Vol.* 4). Cambridge: Cambridge University Press.

[ref4] Bryant E. M. (2008). Real lies, white lies, and gray lies: towards a typology of deception. Kaleidoscope 7, 23–48.

[ref5] Cameron C. A. Lau C. Fu G. Lee K. (2012). Development of children’s moral evaluations of modesty and self-promotion in diverse cultural settings. J. Moral Educ 41, 61–78. doi: 10.1080/03057240.2011.617414, PMID: 23606783 PMC3628628

[ref6] Chan S. M. Bowes J. Wyver S. (2009). Parenting style as a context for emotion socialization. Early Educ. Dev. 20, 631–656. doi: 10.1080/10409280802541973

[ref7] Chen H. Cohen P. Chen S. (2010). How big is a big odds ratio? Interpreting the magnitudes of odds ratios in epidemiological studies. Commun. Stat. Simulat. Comput. 39, 860–864. doi: 10.1080/03610911003650383

[ref8] Chiu Loke I. Heyman G. D. Itakura S. Toriyama R. Lee K. (2014). Japanese and American children’s moral evaluations of reporting on transgressions. Dev. Psychol. 50, 1520–1531. doi: 10.1037/a0035993, PMID: 24588520

[ref9] Cozma I. (2011). How are individualism and collectivism measured. Rom. J. Appl. Psychol. 13, 11–17.

[ref11] Elster A. Gelfand M. J. (2021). When guiding principles do not guide: The moderating effects of cultural tightness on value-behavior links. J. Pers. 89, 325–337. doi: 10.1111/jopy.12584 32772368

[ref12] Faul F. Erdfelder E. Buchner A. Lang A.-G. (2009). Statistical power analyses using G*Power 3.1: Tests for correlation and regression analyses. Behav. Res. Methods 41, 1149–1160. doi: 10.3758/BRM.41.4.1149, PMID: 19897823

[ref13] Fu G. Brunet M. K. Lv Y. Ding X. Heyman G. D. Cameron C. A.. (2010). Chinese children's moral evaluation of lies and truths—roles of context and parental individualism–collectivism tendencies. Infant Child Dev. 19, 498–515. doi: 10.1002/icd.680, PMID: 21072133 PMC2975357

[ref14] Fu G. Luo Y. C. Heyman G. D. Wang B. Cameron C. A. Lee K. (2016). Moral evaluations of lying for one's own group. Infant Child Dev. 25, 355–370. doi: 10.1002/icd.1941

[ref15] Gelfand M. J. Nishii L. H. Raver J. L. (2006). On the nature and importance of cultural tightness-looseness. J. Appl. Psychol. 91, 1225–1244. doi: 10.1037/0021-9010.91.6.1225 17100480

[ref16] Gelfand M. J. Raver J. L. Nishii L. Leslie L. M. Lun J. Lim B. C.. (2011). Differences between tight and loose cultures: A 33-nation study. Science 332, 1100–1104. doi: 10.1126/science.1197754, PMID: 21617077

[ref18] Hentschel M. Averbeck B. B. Lange-Küttner C. (2022). The role of IQ and social skills in coping with uncertainty in 7-to 11-year-old children. Zeitschrift für Entwicklungspsychologie und Pädagogische Psychologie 54, 105–123. doi: 10.1026/0049-8637/a000256

[ref19] Heyman G. D. Ding X. P. Fu G. Xu F. Compton B. J. Lee K. (2020). Young children selectively hide the truth about sensitive topics. Cogn. Sci. 44:e12824. doi: 10.1111/cogs.12824, PMID: 32180270

[ref20] Hsieh F. Y. Bloch D. A. Larsen M. D. (1998). A simple method of sample size calculation for linear and logistic regression. Stat. Med. 17, 1623–1634. doi: 10.1002/(SICI)1097-0258(19980730)17:14<1623::AID-SIM871>3.0.CO;2-S, PMID: 9699234

[ref22] Lau Y. L. Cameron C. A. Chieh K. M. O’Leary J. Fu G. Lee K. (2013). Cultural differences in moral justifications enhance understanding of Chinese and Canadian children’s moral decisions. J. Cross-Cult. Psychol. 44, 461–477. doi: 10.1177/0022022112453315

[ref23] Lee K. Cameron C. A. Xu F. Fu G. Board J. (1997). Chinese and Canadian children’s evaluations of lying and truth telling: Similarities and differences in the context of pro- and anti-social behaviors. Child Dev. 68, 924–934. doi: 10.1111/j.1467-8624.1997.tb01971.x, PMID: 29106719

[ref25] Levine E. E. Lupoli M. J. (2022). Prosocial lies: Causes and consequences. Curr. Opin. Psychol. 43, 335–340. doi: 10.1016/j.copsyc.2021.08.006, PMID: 34537461

[ref26] Ma F. Xu F. Heyman G. D. Lee K. (2011). Chinese children’s evaluations of white lies: Weighing the consequences for recipients. J. Exp. Child Psychol. 108, 308–321. doi: 10.1016/j.jecp.2010.08.015, PMID: 20951996 PMC2991529

[ref27] Mojdehi A. S. Shohoudi A. Talwar V. (2020). Children’s moral evaluations of different types of lies and parenting practices and across cultural contexts. Curr. Psychol. 41, 5420–5433. doi: 10.1007/s12144-020-01059-7

[ref28] Pearson E. Rao N. (2003). Socialization goals, parenting practices, and peer competence in Chinese and English preschoolers. Early Child Dev. Care 173, 131–146. doi: 10.1080/0300443022000022486

[ref29] Popliger M. Talwar V. Crossman A. (2011). Predictors of children’s prosocial lie-telling: Motivation, socialization variables, and moral understanding. J. Exp. Child Psychol. 110, 373–392. doi: 10.1016/j.jecp.2011.05.003, PMID: 21663918

[ref30] Robinson C. C. Mandleco B. Olsen S. F. Hart C. H. (1995). Authoritative, authoritarian, and permissive parenting practices: Development of a new measure. Psychol. Rep. 77, 819–830. doi: 10.2466/pr0.1995.77.3.819

[ref31] Saltzstein H. D. (1994). The relation between moral judgment and behavior: A social-cognitive and decision-making analysis. Hum. Dev. 37, 299–312. doi: 10.1159/000278274

[ref32] Singelis T. M. Triandis H. C. Bhawuk D. P. Gelfand M. J. (1995). Horizontal and vertical dimensions of individualism and collectivism: A theoretical and measurement refinement. Cross-Cult. Res. 29, 240–275. doi: 10.1177/106939719502900302

[ref33] Talwar V. Lee K. (2002). Emergence of white-lie telling in children between 3 and 7 years of age. Merrill Palmer Q. 48, 160–181. doi: 10.1353/mpq.2002.0009

[ref34] Talwar V. Lee K. Bala N. Lindsay R. C. L. (2002). Children's conceptual knowledge of lying and its relation to their actual behaviors: Implications for court competence examinations. Law Hum. Behav. 26, 395–415. doi: 10.1023/A:1016379104959, PMID: 12182530

[ref35] Talwar V. Murphy S. M. Lee K. (2007). White lie-telling in children for politeness purposes. Int. J. Behav. Dev. 31, 1–11. doi: 10.1177/0165025406073530, PMID: 18997880 PMC2581483

[ref36] Warneken F. Orlins E. (2015). Children tell white lies to make others feel better. Br. J. Dev. Psychol. 33, 259–270. doi: 10.1111/bjdp.12083, PMID: 25773019

