# Supplementary material for: Prosocial lie-telling in preschoolers: The impacts of ethnic background, parental factors, and perceived consequence for the partner
Source: Front Psychol. 2023 Mar 30;14:1128685. doi: 10.3389/fpsyg.2023.1128685 (PMC10098184; doi:10.3389/fpsyg.2023.1128685)
Supplement: Supplementary file 1 [file Table_1.docx]

**Supplementary Table S1**

*Sociodemographic Characteristics of Participants*

|  | European Canadian (*n* = 49) | | Chinese Canadian (*n* = 45) | | Total (*N* = 94) | |
| --- | --- | --- | --- | --- | --- | --- |
|  | *n* | % | *n* | % | *N* | % |
| *Gender* |  |  |  |  |  |  |
| Female | 27 | 55.1 | 29 | 64.4 | 56 | 59.6 |
| Male | 22 | 44.9 | 16 | 35.6 | 38 | 40.4 |
| *Highest parental educational level* | | | | | | |
| No diploma | 1 | 2.0 | 0 | 0.0 | 1 | 1.1 |
| Diploma | 5 | 10.2 | 0 | 0.0 | 5 | 5.3 |
| College | 6 | 12.2 | 2 | 4.4 | 8 | 8.5 |
| University | 26 | 53.1 | 31 | 68.9 | 57 | 60.6 |
| Graduate | 11 | 22.4 | 11 | 24.4 | 22 | 23.4 |
| NR | 0 | 0.0 | 1 | 2.2 | 1 | 1.1 |
| *Family income* |  |  |  |  |  |  |
| >50K | 5 | 10.2 | 2 | 4.4 | 7 | 7.4 |
| 50-100K | 10 | 20.4 | 10 | 22.2 | 20 | 21.3 |
| 100-150K | 14 | 28.6 | 16 | 35.6 | 30 | 31.9 |
| 150-200K | 11 | 22.4 | 10 | 22.2 | 21 | 22.3 |
| 200K+ | 9 | 18.4 | 6 | 13.3 | 15 | 16.0 |
| NR | 0 | 0.0 | 1 | 2.2 | 1 | 1.1 |
| *Type of neighborhood* | | | | | | |
| Urban | 23 | 46.9 | 19 | 42.2 | 42 | 44.7 |
| Suburban | 24 | 49.0 | 23 | 51.1 | 47 | 50.0 |
| Rural | 2 | 4.1 | 2 | 4.4 | 4 | 4.3 |
| NR | 0 | 0.0 | 1 | 2.2 | 1 | 1.1 |

*Note***.** Participants were on average 4.9 years old (*SD* = .520), and participant age did not differ significantly by ethnic group or condition. Percentages do not always add up to 100 due to rounding. NR = No response.
