# Supplementary material for: Prosocial lie-telling in preschoolers: The impacts of ethnic background, parental factors, and perceived consequence for the partner
Source: Front Psychol. 2023 Mar 30;14:1128685. doi: 10.3389/fpsyg.2023.1128685 (PMC10098184; doi:10.3389/fpsyg.2023.1128685)
Supplement: Supplementary file 2 [file Table_2.docx]

**Supplementary Table S2**

*Children’s Prosocial Lying Across Tasks in Relation to All Variables of Interest: GEE Results*

| Predictor | *B* | S.E. | Wald | *df* | Sig. |  | 95% CI for *B*  [LL, UL] |
| --- | --- | --- | --- | --- | --- | --- | --- |
| (Intercept) | -4.580 | 3.359 | 1.860 | 1 | .173 |  | [-11.16, 2.00] |
| Ethnic Group | 0.192 | 0.647 | 0.088 | 1 | .766 |  | [-1.08, 1.46] |
| Condition | -0.438 | 0.618 | 0.502 | 1 | .479 |  | [-1.65, 0.77] |
| Ethnic Group x Condition | 0.534 | 0.872 | 0.374 | 1 | .541 |  | [-1.18, 2.24] |
| Authoritativeness | 1.740 | 0.549 | 10.050 | 1 | .**002**** |  | [0.66, 2.82] |
| Authoritarianism | -0.263 | 0.453 | 0.339 | 1 | .561 |  | [-1.15, 0.62] |
| Permissiveness | -0.515 | 0.506 | 1.034 | 1 | .309 |  | [-1.51, 0.48] |
| Horizontal Individualism | 0.204 | 0.144 | 2.010 | 1 | .156 |  | [-0.08, 0.49] |
| Vertical Individualism | 0.416 | 0.240 | 3.005 | 1 | .083 |  | [-0.05, 0.89] |
| Horizontal Collectivism | -0.374 | 0.249 | 2.257 | 1 | .133 |  | [-0.86, 0.11] |
| Vertical Collectivism | -0.036 | 0.182 | 0.038 | 1 | .845 |  | [-0.39, 0.32] |

*Note.* *B* represents unstandardized regression weights. *S.E.* represents standard error. *LL* and *UL* indicate the lower and upper limits of a confidence interval, respectively. The reference category for ethnic group is “Chinese.” The reference category for condition is “consequence.” ***p* < .01.
