# Supplementary material for: Prosocial lie-telling in preschoolers: The impacts of ethnic background, parental factors, and perceived consequence for the partner
Source: Front Psychol. 2023 Mar 30;14:1128685. doi: 10.3389/fpsyg.2023.1128685 (PMC10098184; doi:10.3389/fpsyg.2023.1128685)
Supplement: Supplementary file 4 [file Image_1.pdf]

Supplementary Figure S1

Stimuli Used in the Art Rating Task

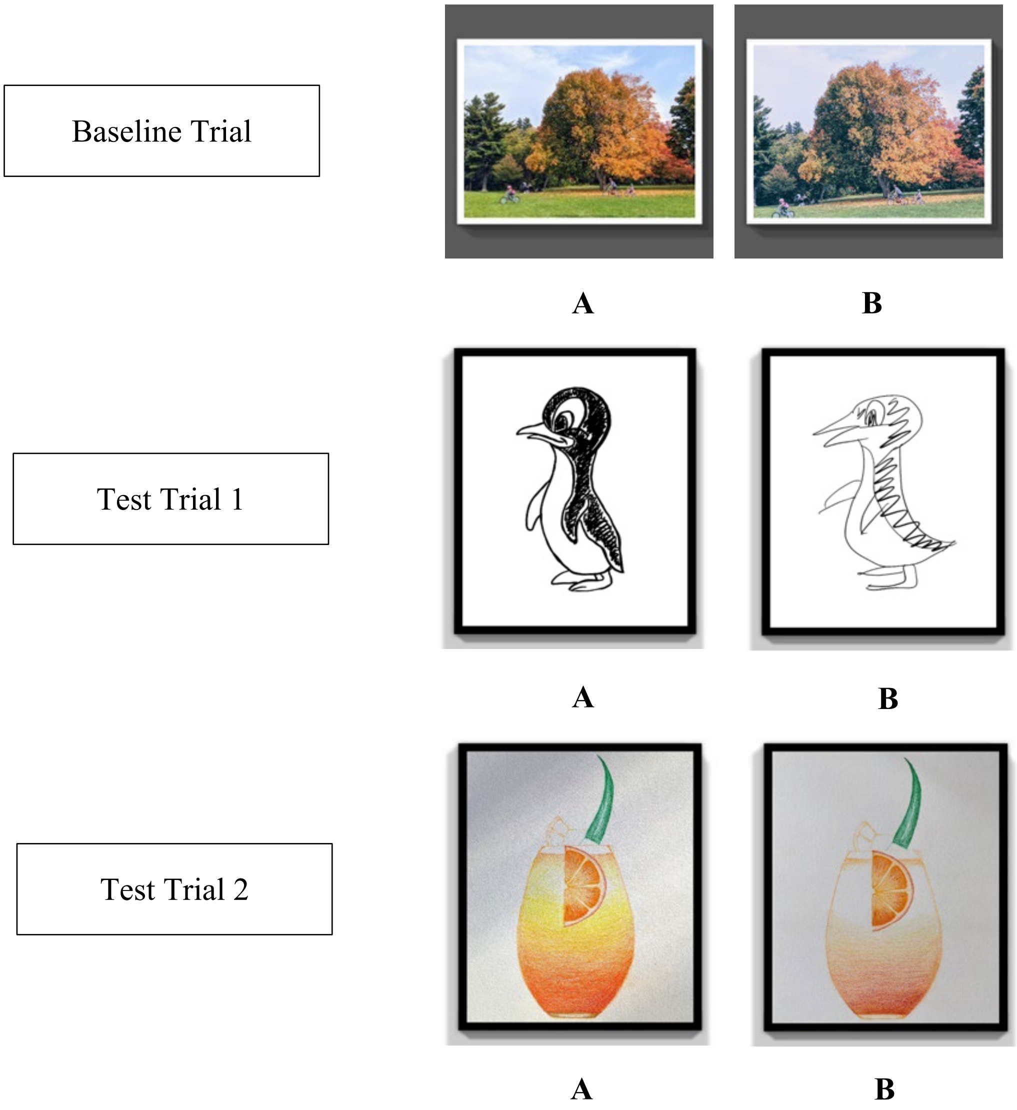

Note. In each trial, A represents the better artwork, while B represents the worse artwork.
